# Immunotherapy-Associated Pancreatic Adverse Events: Current Understanding of Their Mechanism, Diagnosis, and Management

**DOI:** 10.3389/fonc.2021.627612

**Published:** 2021-02-25

**Authors:** Ya Liu, Hao Zhang, Li Zhou, Weichun Li, Le Yang, Wen Li, Kezhou Li, Xubao Liu

**Affiliations:** ^1^ Department of Pancreatic Surgery, West China Hospital, Sichuan University, Chengdu, China; ^2^ Core Facilities, West China Hospital, Sichuan University, Chengdu, China; ^3^ CAAC Academy, Civil Aviation Flight University of China, Guanghan, China; ^4^ Lung Cancer Center, West China Hospital, Sichuan University, Chengdu, China

**Keywords:** immune checkpoint inhibitors, immune-related adverse events, ICIs associated with diabetes mellitus, amylase/lipase, pancreatitis, pancreatic exocrine insufficiency

## Abstract

Immune checkpoint inhibitors (ICIs) such as anti-programmed death-1 (PD-1) and its ligand PD-L1 and anti-cytotoxic T-lymphocyte antigen 4 (CTLA-4) monoclonal antibodies, are involved in T cell-mediated immune response augmentation and promote anti-tumor immunity. Cancer patients treated with combination of immunotherapy, chemotherapy, radiotherapy, and targeted therapy exhibit superior clinical outcomes and tolerance compared with patients treated with monotherapies. However, immutherapy is associated with several concomitant immune-related adverse events (irAEs). For instance, IrAEs interferes with function of gastrointestinal tract, endocrine, dermatological, nervous system and musculoskeletal systems. ICIs-associated pancreatic injury might causes decrease in endocrine and exocrine pancreatic function, resulting in metabolic and nutritional disorders. Clinicians who administer immune checkpoint inhibitors to cancer patients are diagnosed with hyperglycemia, abdominal pain and steatorrhea. Currently, the precise mechanism of ICIs-associated pancreatic injury has not been fully explored. This paper summarizes incidence, diagnosis, clinical characteristics, potential mechanisms, and treatment management patterns of ICIs-associated pancreatic AEs based on previous studies. In addition, possible management approaches of these adverse effects are presented in this paper. in the findings summarized in this paper lay a basis for management of ICIs-associated pancreatic AEs and expanding future immunotherapy applications.

## Introduction

**Figure 1:**
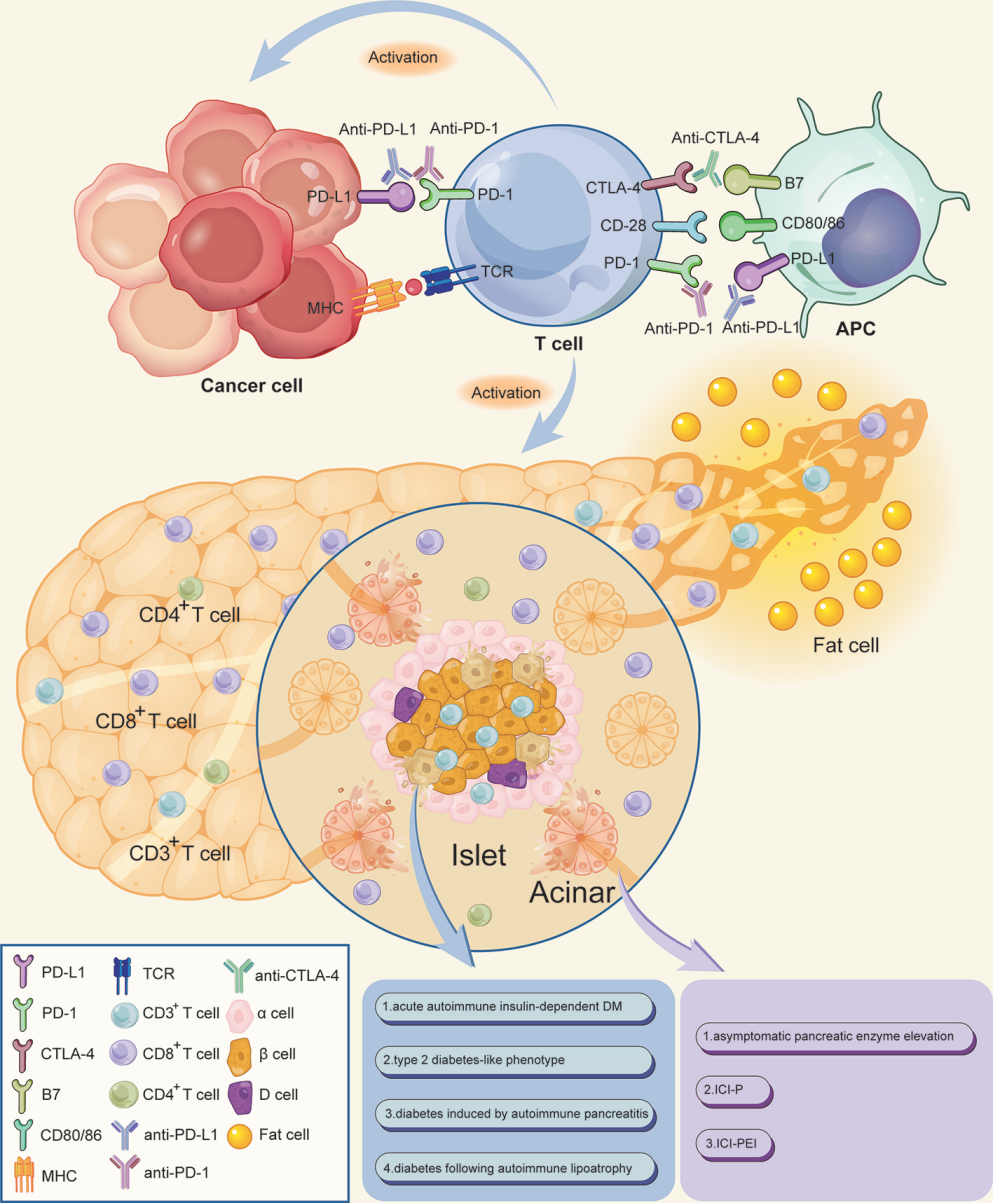
Mechanism of ICIs associated pancreatic adverse events. Anti-PD-1/PD-L1 and anti-CTLA-4 monoclonal antibodies bind and block inhibitory signals, thus causing the widespread activation and expansion of T lymphocytes by blocking the inhibitory signals of T cells and enhancing the ability of the immune system to fight cancer cells. CD3+ T lymphocytes densely infiltrate the pancreatic islets, thereby increasing the ratio of CD8+/CD4+ T lymphocytes in peritumoral areas. The increased CD8+ T cells may cause damage to pancreatic cells, including islet β-cells and acinar, thereby destroying exocrine and endocrine pancreatic tissues which lead to a decrease in endocrine and exocrine pancreatic function. Meanwhile, a small proportion of patients would accompanied by peripancreatic fatty infiltration and pancreatic atrophy. APC, antigen-presenting cell; MHC, major histocompatibility complex; TCR, T cell receptor.

Programmed death 1 (PD-1) receptors bind to programmed death-ligand 1 (PD-L1), transport negative signals to T cells, and regulate functions of effector T cells. These receptors are expressed by T cells, B cells, and natural killer cells. In addition to normal T cells, several tumor cells upregulate PD-L1 on their surface, thus evading antitumor immune response and promoting immune tolerance by inactivating T cells through the PD-1/PD-L1 axis. These processes cause delay in the immune activation cycle. Cytotoxic T lymphocyte antigen 4 (CTLA-4) expressed on the surface of T cells downregulates immune responses against cancer cells in the early stages of the immune activation cycle. The mechanism of action is by interacting with the surface molecules B7.1 (CD80) and B7.2 (CD86) on antigen-presenting cells (1–5). These interactions promote tumor cells and aid in evasion of immunosurveillance. Therefore, use of immune checkpoint inhibitors (ICIs), including anti-PD-1 monoclonal antibodies (nivolumab and pembrolizumab), anti-PD-L1 monoclonal antibodies (atezolizumab, avelumab, and durvalumab), and anti-CTLA-4 monoclonal antibodies (ipilimumab and tremelimumab), triggers activation and expansion of T lymphocytes. These inhibitors act by blocking inhibitory signals of T cells and enhancing ability of the immune system to fight cancer cells (**Figure 1**).

Mechanisms of the 3 immunotherapy agents are different from each other and ICIs alone or in combination with chemotherapy are conventional first- or second-line therapies. This has been attributed to favorable survival durations and tolerance for many types of malignancies, including metastatic melanoma (6), advanced non-small cell lung cancer (7), Hodgkin lymphoma (8), metastatic renal cell carcinoma (9), and unresectable hepatocellular carcinoma (10). Despite the efficacy of immunotherapy, immune-related adverse events (irAEs), often involving endocrine tissues including pneumonitis, hypophysitis, thyroiditis, colitis, pancreatitis, and autoimmune diabetes, have emerged as potential challenges to patients (11, 12).

Incidence rate of ICIs-associated pancreatic AEs (pancreatitis, hyperglycemia, elevated amylase/lipase, and exocrine pancreatic insufficiency) is relatively low. Most of these events have been described as a case report (**Table 1**). Cancer patients treated with ICIs present with symptoms collectively described as ICIs-associated pancreatic AEs including abdominal pain, vomiting, dyspepsia, irregular stools, and large daily glucose fluctuations (7, 13, 32). ICIs-associated pancreatic AEs are rare, however they result in poor quality of life and affect safety of patients. This minireview retrieved data on ICIs-associated pancreatic AEs from several publications and summarizes different clinical and pathophysiological mechanisms and effective treatments for these effects (**Table 2**).

**Table 1:** Published case reports and case series of immune checkpoint inhibitors associated with diabetes mellitus.

Authors	Patient	Cancer type	RelevantHistory	Agent	Time to onset (weeks)	Presenting features				Autoantibodies	HLA	Treatment	Discontinued ICI	Other irAEs	Outcome
						Clinical	Glucose(mmol/liter)	C-peptide	HbA1c(%)						
Kusuki et al. (13)	72 M	NSCLC	Colon cancer	Pembrolizumab	16	DKA	27.1	0.39 ng/ml	8.1	NR	DRB1*09:01-DQB1*03:03	NR	Yes	None	CR
Porntharukchareon et al. (7)	70 M	NSCLC	None	Pembrolizumab + Ipilimumab	14	DKA	44.1	< 0.1 ng/ml	6.5	None	NR	Pembrolizumab (200 mg q3w), Ipilimumab (1 mg/kg q6w)	Yes	Isolated adrenocorticotropic hormone deficiency	NR
Singh et al. (9)	65 F	RCC	None	Ipilimumab + Nivolumab	6	DKA	33.5	0.1 mg/dl	8.5	None	NR	Ipilimumab (55 mg × 4 cycles = 220 mg), Nivolumab (160 mg × 4 mg = 640 mg) q3w	Yes	Hypothyroidism	NR
Wen et al. (10)	56 M	HCC	Chronic viral hepatitis B	Sintilimab	24	DKA	22.2	1.12 ng/ml	7.8	None	A∗0201	200 mg q3w	Yes	None	PR
Yilmaz et al. (14)	49 M	RCC	None	Nivolumab	40	DKA	44.4	0.24 nmol/liter	10.9	None	NR	3 mg/kg q2w	Yes	NR	NR
Falcao et al. (15)	57 F	RCC	Type 2 DM	Nivolumab	8	Hyperglycemia	NR	NR	10.5	NR	NR	3 mg/kg q2w	No	Autoimmune lipoatrophy, Hepatitis	PD
Kotwal et al. (16)	M (12) F (9)	Lung cancer (5) Melanoma (9) Breast cancer (2) RCC (1) Multiple myeloma (1) Lymphoma (1) MCC (1) Esophageal cancer (1) Pancreatic cancer (1)	NR	Pembrolizumab	8.4	DKA	32.2	0.9 ng/ml	8.6	GAD	NR	NR	NR	Thyroiditis	NR
			NR	Pembrolizumab	8.8	DKA	53.9	<0.1 ng/ml	10.7	GAD				Thyroiditis	
			NR	Pembrolizumab	2.4	DKA	54	<0.1 ng/ml	7.8	GAD/IA-2/IAA				Thyroiditis	
			NR	Pembrolizumab	19.2	Hyperglycemia	33.3	0.3 ng/ml	10.5	GAD/IAA				NR	
			NR	Nivolumab	22	Hyperglycemia	33.3	NR	NR	NR				Thyroiditis	
			NR	Ipilimumab → Pembrolizumab	19.6	Hyperglycemia	20.9	5.2 ng/ml	9.7	NR				NR	
			NR	Pembrolizumab	31.2	DKA	35.8	NR	9.7	None				Thyroiditis, Hypophysitis	
			NR	Pembrolizumab	38.8	DKA	25.6	0.7 ng/ml	7.8	GAD				NR	
			NR	Pembrolizumab	94.4	DKA	23.8	NR	11.3	NR				Dermatitis, Hypophysitis, Arthritis	
			NR	Pembrolizumab	12	DKA	23.7	NR	11.2	NR				NR	
			NR	Pembrolizumab	2.8	DKA	77.2	0.4 ng/ml	8.8	NR				NR	
			NR	Pembrolizumab	32.8	DKA	24.9	NR	10.6	None				NR	
			Type 2 DM	Nivolumab	15.6	Hyperglycemia	32.2	NR	10	NR				Thyroiditis	
			Type 2 DM	Nivolumab	22.8	Hyperglycemia	21	NR	10	NR				Dermatitis, Adrenal ansufficiency	
			Type 2 DM	Pembrolizumab	20	Hyperglycemia	13.3	NR	8.6	NR				Thyroiditis	
			Type 2 DM	Pembrolizumab	15.2	Hyperglycemia	20.4	NR	11.3	NR				Ocular	
			Type 2 DM	Pembrolizumab	5.6	Hyperglycemia	11.6	NR	9	NR				NR	
			Type 2 DM	Pembrolizumab	27.6	Hyperglycemia	13.8	NR	8.1	NR				Thyroiditis, Hypophysitis	
			Type 2 DM	Ipilimumab → Pembrolizumab	49.2	Hyperglycemia	16.9	NR	11.8	NR				Hepatitis	
			Type 2 DM	Nivolumab	7.2	Hyperglycemia	10	NR	9.5	NR				Thyroiditis	
			Type 2 DM	Pembrolizumab	6.8	Hyperglycemia	55.5	NR	12	NR				NR	
Hakami et al. (17)	52 M	Melanoma	None	Pembrolizumab	23	DKA	38.6	<0.01µg/liter	8.3	None	NR	2 mg/kg q3w	No	Hypothyroidism	NR
Mengíbar et al. (18)	55 M	Urothelial cancer	Asymptomatic subclinical hyperthyroidism	Durvalumab	3	DKA	23.2	0.02 ng/ml	8.4	GAD/IA-2	NR	10 mg/kg	Yes	Primary autoimmune hypothyroidism	NR
Okahata et al. (19)	52 M	Breast cancer	None	Nivolumab	8	F1DM	22.3	0.3 ng/mL	7.8	None	DRB1* 14:05, 14:06 DQB1* 03:01, 03:03	0.36 mg/kg q2w	NR	None	NR
Sakaguchi et al. (20)	68 F	Melanoma	Graves disease	Nivolumab	81	DKA	17.4	0.2 nmol/liter	8.2	None	DRB1*09:01	3 mg/kg q3w	NR	Thyrotoxicosis	NR
Shibayama et al. (21)	81 F	MCC	None	Avelumab	20	F1DM	26.8	1.07 ng/ml	7.5	GAD/IA-2	DRB1*09:01:02, DRB1*14:54:01, DQA1*01:04, DQA1*03:02, DQB1*05:02:01, DQB1*03:03:02	523 mg q2w	Yes	None	NR
Marchand et al. (22)	55 M	Pleomorphic carcinoma	None	Nivolumab	9 cycles	DKA	NR	Undetectable	8.2	None	None	NR	NR	Hypophysitis, Pancreas atrophy	NR
Gauci et al. (6)	73 M	Melanoma	None	Nivolumab	6	DKA	27.78	Undetectable	7.2	GAD/ZnT8/IA-2	NR	NR	Yes	NR	NR
	84 F	Melanoma	None	Pembrolizumab	6	DKA	26.71	NR	8.6	GAD/ZnT8/IA-2	NR	NR	Yes	NR	NR
	34 M	Melanoma	None	ipilimumab + Nivolumab	11	DKA	21.44	NR	5.4	None	NR	NR	No	NR	NR
Li et al. (23)	67 M	NSCLC	None	Pembrolizumab	< 7	DKA	26.98	0.51 ng/ml	8	None	NR	2 mg/kg q3w	Yes	NR	NR
Dehghani et al. (24)	63 M	Melanoma	None	Nivolumab	72	Hyperglycemia	11	0.41 nmol/liter	9.3	None	NR	NR	Yes	Pancreatitis, Pancreatic atrophy	PD
Matsuura et al. (25)	78 M	NSCLC	Type 2 DM	Nivolumab	5	Asymptomatic	527	<0.1 ng/ml	6.1	GAD	None	3 mg/kg q2w	Yes	NR	NR
Zaied et al. (26)	70 M	RCC	None	Nivolumab	6	DKA	48.8	0.4 ng/ml	8.4	None	NR	3 mg/kg q2w	Yes	None	Death
Shiba et al. (27)	80 F	Melanoma	None	Ipilimumab	4	F1DM	35.5	<0.01 ng/ml	7.7	IAA	DR4	3 mg/kg	NR	None	NR
Takahashi et al. (28)	74 F	Melanoma	None	Nivolumab	17	F1DM	31.7	Urinary C-peptide <0.6 μg/day	8.0	None	NR	2 mg/kg q3w	Yes	None	NR
Sakurai et al. (29)	68 F	RCC	None	Nivolumab	14	Hyperglycemia	26.3	0.24 ng/ml	6.9	None	DRB1*09:01, DQB1*03:03	3 mg/kg q2w	No	Subclinical Hypothyroidism, Adrenal insufficiency	NR
Capitao et al. (30)	74 F	Lung cancer	Arterial hypertension, Hypercholesterolaemia	Nivolumab	5	DKA	58.9	<0.01 ng/ml	8.7	GAD	DRB1*04	3 mg/kg q2w	No	Dehydration, Acute kidney injury	PR
Kumagai et al. (31)	73 M	NSCLC	None	Nivolumab	25	Hyperglycemia	>1111.1	0.49 ng/ml	13.4	None	DRB1*09:03-DQB1*03:03 DRB1*01:01-DQB1*05:01	3 mg/kg q2w	NR	Pneumonitis, Vitiligo	NR
Changizzadeh et al. (4)	42 M	Melanoma	None	Nivolumab + Ipilimumab.	12	DKA	40.4	NR	6.5	None	NR	Ipilimumab (3 mg/kg), Nivolumab (1 mg/kg) q3w	NR	Immune-mediated colitis	NR
Godwin et al. (32)	34 F	NSCLC	None	Nivolumab	4	DKA	739	<0.1 ng/ml	7.1	GAD/IA-2/IAA	A30:01,30:02 (A30) D09:CTZ,09:CTZ (DR9)	3 mg/kg q2w	Yes	NR	CR
Hickmott et al. (1)	57 M	Urothelial cancer	None	Atezolizumab	15	DKA	24	0.65 ng/ml	7.5	None	DRB1*04, DQB1*03	NR	No	No	Death
Munakata et al. (8)	72 M	Hodgkin lymphoma	None	Nivolumab	12	F1DM	20.8	Urinary C-peptide 5.0 μg/day	7.3	None	B*4002	3 mg/kg q2w	Yes	Pancreatitis	PD
Chae et al. (33)	76 M	NSCLC	NR	Pembrolizumab	3	Asymptomatic	34.2	0.81 ng/ml	6.3	GAD/IA-2	NR	NR	No	NR	NR
Kapke et al. (34)	83 M	HNSCC	Hypothyroidism	Nivolumab	12	DKA	23.7	0.32 ng/ml	7.4	GAD	DRB1*08, DRB1*11, DQB1*03, DQB1*04, DQA1*04, and DQA1*05	240 mg q2w	Yes	Diffuse colitis	NR
	63 M	Urothelial Carcinoma	Hypothyroidism	Atezolizumab	24	DKA	44.5	0.02 ng/ml	7.8	GAD	DRB1*03, DRB1*04, DQB1*02, DQB1*03, DQA1*03, and DQA1*05	200 mg q3w	NR	Cardiac tamponade, Distal tracheal narrowing	Death
Teramoto et al. (35)	63 F	Melanoma	None	Nivolumab	30	DKA	36.7	<0.1 ng/ml	8.9	None	NR	2 mg/kg q3w	Yes	NR	PD
Ishikawa et al. (36)	54 F	Melanoma	None	Nivolumab	40	Hyperglycemia	32.2	1 ng/ml	7	None	HLA-B*15:01,*40:06, DRB1*04:05,*04:06, DQB1 *03:02, and *04:01	2 mg/kg q3w	No	NR	SD
Usui et al. (37)	31 M	NSCLC	None	Nivolumab	<2	DKA	40.8	<0.03 ng/ml	6.4	GAD	DRB1*04:05-DQB1*04:01	NR	NR	NR	NR
	62 F	NSCLC	None	Nivolumab	10	Hyperglycemia	13.7	Urinary C-peptide 2.6 µg/day	6.5	None	DRB1*09:01-DQB1*03:03	NR	NR	NR	NR
Mizab et al. (38)	58 M	Melanoma	None	Pembrolizumab	12	F1DM	33.4	0.007 nmol/l	7.4	None	NR	2 mg/kg q3w	NR	NR	NR
Leonardi et al. (39)	66 M	NSCLC	None	Pembrolizumab	20	DKA	35.3	0.3 ng/ml	7.6	GAD	NR	2 mg/kg q3w	No	NR	NR
Hofmann et al. (40)	58 F	Melanoma	None	Pembrolizumab	3	Hyperglycemia	NR	Low	NR	GAD/IA2	NR	NR	Yes	NR	SD
	78 F	Melanoma	Type 2 DM	Ipilimumab _+_ Nivolumab	3	DKA	NR	Low	NR	GAD	NR	NR	NR	Vomiting, diarrhea	SD
	70 F	Melanoma	None	Nivolumab	6	Hyperglycemia	NR	<16 pmol/l	NR	None	NR	NR	No	NR	CR
Okamoto et al. (41)	55 F	Melanoma	None	Nivolumab	48	F1DM	32	1.0 ng/ml	7.0	None	DRB1*04, DQB1*04	2 mg/kg q3w	Yes	Pancreas atrophy	NR
Miyoshi et al. (42)	66 F	Melanoma	None	Nivolumab	16	F1DM	29	0.23 ng/ml	7.3	None	DQB1*03	2 mg/kg q3w	No	None	NR
Hansen et al. (43)	58 M	Melanoma	NR	Pembrolizumab	51	F1DM	22.2	NR	9.7	GAD	NR	2 mg/kg q3w	No	Hypothyroidism, Fatigue, Hair depigmentation, Gastroesophageal reflux	SD
Aleksova et al. (44)	60 M	Melanoma	None	Ipilimumab → Pembrolizumab	5	DKA	27	57 pmol/liter	7.1	None	NR	Ipilimumab (3 mg/kg, 4 cycles), Pembrolizumab (2 mg/kg, 5 weeks)	Yes	None	NR
Humayun et al. (45)	55 M	Melanoma	None	Ipilimumab	9 cycles	F1DM	42	NR	10.7	None	NR	NR	Yes	Hypopituitarism	NR
Martin-Liberal et al. (46)	54 F	Melanoma	Asthma	Ipilimumab → Pembrolizumab	9	DKA	NR	NR	NR	GAD	DRB1*04, DQB1*0302	Ipilimumab (3 mg/kg, 4 cycles), Pembrolizumab (2 mg/kg, 3 cycles)	No	NR	PR
Hughes et al. (47)	55 F	Melanoma	Autoimmune thyroid disease	Nivolumab	20	DKA	30	<0.1 ng/ml	6.9	NR	A2.1+, DR4+	NR	NR	NR	NR
	83 F	NSCLC	None	Nivolumab	4	DKA	29	<.0.1 ng/ml	7.7	GAD	A2.1+, DR4+	NR	NR	NR	NR
	63 M	RCC	Hypertension	Nivolumab	16	Hyperglycemia	19	1.3 ng/ml	8.2	GAD/ICA/IAA	A2.1+, DR4+	NR	NR	NR	NR
	58 M	SCLC	Type 2 diabetes	Nivolumab	1	DKA	42	<0.1 ng/ml	9.7	GAD	A2.1+	NR	NR	NR	NR
	64 F	Melanoma	Autoimmune thyroid disease, Psoriasis	Pembrolizumab	< 4	DKA	39	0.5 ng/ml	7.4	NR	DR4+	NR	NR	NR	NR
Mellati et al. (48)	70 M	Lung cancer	NR	PDL-1 inhibitor (not named)	15	DKA	23	0.3 ng/ml	9.8	None	NR	NR	NR	NR	Death
	66 F	SCC Jaw	NR	PDL-1 inhibitor (not named)	7	DKA	42	<0.1 ng/ml	9.4	GAD	DR3-DQ2, DR4-DR8	NR	NR	NR	NR
Gaudy et al. (49)	44 F	Melanoma	Autoimmune thyroid disease	Pembrolizumab	5	DKA	50	Undetectable	6.85	None	NR	NR	Yes	Acute renal failure	NR

SCC Jaw, Sarcomatoid squamous cell carcinoma of the Jaw; NSCLC, Non-small cell lung cancer; SCLC, Small-cell lung cancer; RCC, Renal cell carcinoma; MCC, Merkel cell carcinoma; HNSCC, Head and neck squamous cell carcinoma; HCC, Hepatocellular carcinoma; Type 2 DM, Type 2 diabetes mellitus; NR, Not reported; DKA, Diabetic ketoacidosis; GAD, Glutamic acid decarboxylase; IA-2, Islet-associated antigen-2; IAA, Insulin autoantibodies; ICA, Islet cell antibody; ZnT8, Zinc transporter 8; F1DM, Fulminant type 1 diabetes; q2w, every two weeks; q3w, every three weeks.

**Table 2:** Immune checkpoint inhibitors associated with pancreatic injury.

Type of pancreatic injury		Characteristics	Possible mechanisms	Treatment	References
Endocrine (ICIs-DM)	Acute autoimmune insulin-dependent DM	Hyperglycemia; FIDM; DKA; Undetectable C-peptide; Autoantibodies	1. β-cells be destroyed by CD8+ T cells, but α-cells are not affected. 2. The period of onset hyperglycemia may linked to the antibodies.	① New-onset hyperglycemia < 200 mg/dl and a history of T2D with low suspicion for DKA should continue ICIs, monitor serial blood glucose and modify diet and lifestyle; and consider endocrine consultation if patients are symptomatic or glucose levels are persistently uncontrolled. ② New-onset fasting glucose > 200 mg/dl, random blood glucose > 250 mg/dl, history of T2D with fasting/random glucose > 250 mg/dl, and workup negative for DKA should be given similar treatments and management. ③ DKA should hold ICIs, require inpatient care and endocrine consultation. DKA should be managed with institutional guidelines: IV fluids +/– potassium supplementation, IV insulin, and hourly monitoring of laboratory indicators (glucose, serum ketones, blood pH, and anion gap) to correct the anion gap and electrolyte disorder.	(6, 37, 50–52)
	Type 2 diabetes-like phenotype	Hyperglycemia; Pre-existing T2DM; Higher BMI; Older age; Hypertension; Detectable C-peptide; Higher HbA1c; CRP; Few with DKA	1. β-cells be destroyed by CD8+ T cells, but α-cells are not affected. 2. T2D-like phenotype can be an insidious side effect on glycemia due to an abnormal chronic subclinical inflammatory state induced by long-term ICIs therapy.		(5, 6, 16, 25, 53, 54)
	Diabetes induced by autoimmune pancreatitis	Hyperglycemia; Higher HbA1c; Pancreatitis; Pancreatic atrophy	1. CD8+ T cells infiltrate in and around the pancreatic islets rather than CD4+ T cells. 2. It cause damage to pancreatic cells, including islet β-cells and acinar, thereby destroying exocrine and endocrine pancreatic tissues and resulting in pancreatitis-related diabetes and pancreatic atrophy.		(22, 24, 55)
	Diabetes following autoimmune lipoatrophy	Hyperglycemia; AGL; Central obesity; Higher HbA1c	1. The histological analysis revealed CD3+ T cells infiltration and extensive fibroelastosis replacement. 2. The worsening of glycemic control is primarily related to the increased IR and concomitant with the progression of autoimmune lipoatrophy.		(15, 55, 56)
Exocrine	Asymptomatic pancreatic enzyme elevation	A mild increase in amylase and lipase levels	1. The relationship between asymptomatically elevated amylase/lipase levels and pancreatitis is still vague. 2. this increase is likely related to T cell-mediated inflammation present in other organs, a metastatic disease, or renal failure, but it is not linked to pancreatic inflammation.	① Pancreatic enzyme monitoring is not recommended as a routine procedure unless pancreatitis is suspected. ② ICIs can be continued.	(57–61)
	ICIs-P	Requires meeting ≥2 criteria: ① significant symptoms of pancreatitis; ② radiographic evidence; ③ changes in laboratory data.	1. CD3+ T lymphocytes densely infiltrate the pancreatic islets in “healthy” areas, and the ratio of CD8+/CD4+ T lymphocytes in “unhealthy” areas increases. It suggests that immune T cell infiltrates may be the prevalent cytotoxic components of ICIs treatment. 2. Dense CD8+, TIA1+, and granzyme B+ lymphoid infiltrate within a biopsied lesion.	① Once ICIs-P is confirmed with the criteria, hospital admission should be recommended. ② For grade 2, ICIs should be discontinued, and 0.5–1 mg/kg/day prednisone/methylprednisolone should be given instead until symptoms improve to grade ≤1. Then, the dose should be tapered for 4–6 weeks, and IV hydration should be administered. ③ For grades 3–4, ICIs should be permanently discontinued, and treatment with a double daily dose of glucocorticosteroids than moderate grade and IV fluids should be provided.	(57, 62–65)
	ICIs-PEI	Abdominal pain; Good appetite; Irregular stools; Steatorrhea; Fecal pancreatic elastase-1 test	CD8+ T cells infiltrate inside and around the pancreas to damage ductal and acinar cells (exocrine pancreas) and even pancreatic atrophy. This alteration decreases the secretion of pancreatic enzymes and affects the release of bicarbonate, water, and enzymes into the duodenum.	PERT	(66, 67)

ICIs, Immune checkpoint inhibitors; DM, Diabetes mellitus; DKA, Diabetic ketoacidosis; FIDM, Fulminant type 1 diabetes; CRP, C-reactive protein; AGL, Acquired generalized lipodystrophy; ICIs-P, ICIs associated with pancreatitis; ICIs-PEI, ICIs associated with pancreatic exocrine insufficiency; PERT, Pancreatic enzyme replacement therapy.

## ICIs Associated With Diabetes Mellitus

The duration before onset of ICIs associated with diabetes mellitus (ICIs-DM) is between 3 weeks and 81 weeks after immunotherapy initiation, and most cases are reported in patients without pre-existing T2DM (11, 25, 47, 68–70). Drug administration significantly changes blood glucose level in cancer patients. HbA1C may be nearly normal or slightly elevated, C-peptide is low or undetectable, and severe cases are associated with diabetic ketoacidosis (DKA) (4, 32). A few ICIs-DM cases with pre-existing T2DM present with rapid hyperglycemia, however, DKA and undetectable C-peptide are uncommon (**Table 1**). Most ICIs-DM cases are observed during treatment with PD-1/PD-L1 inhibitors either alone or in combination with other immunotherapies. On the contrary, a few ICIs-DM cases are reported for patients exposed to CTLA-4 inhibitor monotherapy (7, 11, 70). Patients with advanced tumors are more likely to receive first-line systemic immunotherapy, and may receive combination of PD-1/PD-L1 inhibitors and CTLA-4 inhibitors. ICIs-induced DM incidence of combined therapy is 17%, which is significantly higher compared with that of single PD-1 or PD-L1 inhibitor treatment (6%) (11).

ICIs-DM are classified into 4 types based on the different clinical and biological profiles of ICIs-induced diabetes and their potential pathophysiology. These types include: acute autoimmune insulin-dependent DM, type 2 diabetes-like phenotype, diabetes-induced by autoimmune pancreatitis, and diabetes following autoimmune lipoatrophy (5, 55).

Several studies report that acute autoimmune insulin-dependent DM type, an extremely rare irAE is associated with high mortality. Abrupt onset of hyperglycemia is associated with DKA, whereas hyperglycemia with a concomitant near-normal HbA1c level is an indicator of a fulminant disease. Marie‑Léa Gauci et al., reported that among 132 patients suffering from melanoma, treatment with anti-PD-1 and anti-CTL-4 resulted in T1D accompanied with high blood glucose levels and undetectable C-peptide concentrations in 3 cases. Furthermore, 2 patients presented with positive autoantibodies [glutamic acid decarboxylase (GAD) and tyrosine-phosphatase inhibitor (IA2)] before anti-PD-1 treatment and diabetes onset. These findings imply that these patients are likely to develop T1D (6). Positive autoantibodies are implicated in progressive insulinopenia and insulin-dependent DM, similar to cases of adult-onset autoimmune diabetes (71). CD8+ T cell clones promoted by blocking PD-1 or PD-L1 are the primary cell types involved in destruction of insulin-secreting β-cells associated with DM, without affecting α-cells (6). PD-1/PD-L1 interaction plays a vital role in preventing onset of diabetes in animals. Notably, mice without antibodies develop diabetes, whereas other mice with autoantibodies are unnecessarily predicted to have diabetes (50). Kochupurakkal et al. reported some of the risk factors associated with anti-diabetic immune tolerance. For instance, upregulation of CTLA-4 and regulatory T cells (Treg) through low IL2 production is correlated with high number of DCs and increased T cell stimulation and activation in the absence of negative costimulation induced by PD-1/PD-L1 pathway (51). The period between start of ICIs therapy and onset of T1DM is linked to the presence or absence of GAD antibodies (GADA). Individuals with pre-existing positive GAD antibodies have high risk of developing T1DM in the first 2 months after initiation of treatment. Similarly, GADA-negative patients have a high risk of developing T1DM, however, this effect becomes evident after 2 months of treatment (37). High-risk human leukocyte antigen (HLA) type (HLA-DR4 allele) results in increased susceptibility of humans to T1D (52).

A few subjects exhibit pre-existing T2DM, which is effectively regulated without insulin. These patients experience a sharp increase in blood glucose levels after treatment initiation with ICIs. This outcome implies that ICIs can decompensate glucose control. Most of these patients have a higher BMI, relatively older age, hypertension, detectable C-peptide, and higher HbA1c compared with patients with acute autoimmune insulin-dependent DM. Interestingly, a few cases eventually develop diabetic ketoacidosis since most patients with pre-existing T2DM self administer hypoglycemic drugs, therefore, they self-monitor their blood glucose levels (5, 6, 16, 25, 53). Kotwal et al. reported 9 cases with unexplained worsening of glycemic control for pre-existing T2D after they commenced ICIs therapy. Their HbA1c increased by 10% in 6 months, and most patients needed another antihyperglycemic agent or insulin. Consequently, they had underwent outpatient visits and inpatient admission due to poor blood glucose control (16). Interestingly, laboratory findings showed increased C-reactive protein (CRP) levels in a subset of patients at the time of presentation, implying that immunotherapy induced an inflammatory profile (6). CRP levels are negatively associated with insulin sensitivity, therefore, chronic subclinical inflammation causes insulin resistance (IR) in which is implicated in pathogenesis of T2D (54). Therefore, T2D-like phenotype is an insidious side effect on glycemia attributed to an abnormal inflammatory state induced by long-term ICIs therapy.

Previous studies report several cases of new-onset DM developed autoimmune pancreatitis after cancer immunotherapy. Dehghani et al. reported a 63-year-old Caucasian man without genetic history of diabetes or autoimmune disease, administered with nivolumab for treatment of advanced melanoma. The patient developed focal pancreatitis 15 months after initiation of nivolumab therapy. CT scans showed peripancreatic fatty infiltration around the pancreatic tail. However, he was asymptomatic with normal serum lipase, IgG4, and fasting blood glucose level (6.2 mmol/liter). After 3 more months, his blood lipase was threefold the normal level, fecal elastase-1 decreased to 58 mg/g (normal values > 200 mg/g), serum glucose increased to 11 mmol/liter, HbA1c was 9.3%, and diabetes-associated autoantibodies were negative. Despite these findings, the patent showed no clinical signs. MRI results showed a 50% decrease from the initial pancreatic volume (24). In addition, Marchand et al. reported a dynamic change in pancreatic volume similar to that reported in previous cases. Initially, pancreatic volume increased by 15% after 4 courses of nivolumab, pancreatic atrophy subsequently developed, and the initial volume significantly decreased (63%) 3 months later at the onset of DM (22). Activation of resident immune cells (CD8+ T cell clones) is promoted by immunotherapy infiltrates in and around pancreatic islets rather than CD4+ T cells. This phenomenon explains development of pancreatitis and increase in pancreatic volume before the onset of diabetes. Increased CD8+ T cells might damage pancreatic cells, including islet β-cells and acinar, thereby destroying exocrine and endocrine pancreatic tissues ultimately resulting in pancreatitis-related diabetes and pancreatic atrophy (22, 55).

Autoimmune lipoatrophy also known as acquired generalized lipodystrophy (AGL) causes significant reduction in whole-body fat. Absence of adipocytes promotes ectopic lipid droplet accumulation in other body parts. Abnormal adipose storage is frequently associated with insulin resistance and DM. Falcao et al. reported a case of immune-related AGL. A 57-year-old woman with well-managed T2D (HbA1c 6.7%) received nivolumab for clear cell renal carcinoma. On the 2nd month of treatment, she presented with loss of subcutaneous fat tissues in the facial neck, shoulders, arms, and buttocks. In addition, the patients showed an abnormal pattern of adipose tissues in her abdomen and calves, implying she had central obesity. Further, she was diagnosed with deterioration of glycemic control and HbA1c (10.5%). Her low-density lipoprotein-cholesterol and triglyceride levels increased, whereas the high-density lipoprotein-cholesterol levels decreased. Histological analysis of the subcutaneous biopsy of the medial surface of the arm showed chronic lobular panniculitis with CD3+ lymphocytic infiltration and extensive fibroelastosis replacement. Poor glycemic control was primarily attributed to increased IR and progression of autoimmune lipoatrophy (15, 56). Other studies report new-onset DM secondary to AGL in patients without personal or family history during immunotherapy (15, 55).

Several studies have reported ICIs-associated diabetes providing information for identifying individuals at risk. Routine measurement of HbA1c and blood glucose during treatment should be carried out in clinical work. Furthermore, testing biomarkers including cytokines, novel autoantibodies, and high-risk genetics before ICIs treatment could provide potential predictive valuable information for ICIs-DM (72). Currently, few biomarkers associated with irAE have been reported. Therefore, further studies are essential to identify more potential biomarkers, including proteins, lipids, mRNA, miRNA, and exosomes. National Comprehensive Cancer Network (NCCN) guidelines for management of ICIs-related toxicities (version 1.2020) are the most current comprehensive guidelines adopted in clinical practice, specifically, when symptoms including polyuria, polydipsia, nausea, and vomiting associated with changes in blood glucose levels occur after immunotherapy. Based on these guidelines, patients with (1) new-onset hyperglycemia < 200 mg/dl and (2) history of T2D with low suspicion for DKA should be continuously treated with ICIs. However, their serial blood glucose after administration of each dose should be monitored. The diet and lifestyle of these patients should be modified, and endocrine consultation should be considered if patients are symptomatic or if their glucose levels are persistently uncontrolled. Patients with (3) new-onset fasting glucose > 200 mg/dl, (4) random blood glucose > 250 mg/dl, (5) history of T2D with fasting/random glucose > 250 mg/dl, and (6) workup negative for DKA should be given similar treatments and management. Patients presenting with (7) DKA should have immunotherapy discontinued, however immunotherapy can be restarted once DKA is resolved. Patients requiring inpatient care and endocrine consultation should be administered with insulin as prescribed by a medical oncologist and an endocrinologist. DKA should be managed based on institutional guidelines, i.e., intravenous (IV) fluids +/– potassium supplementation, IV insulin therapy, and hourly monitoring of laboratory indicators (glucose, serum ketones, blood pH, and anion gap) to correct anion gap and electrolyte disorder (73).

## ICIs Associated With Asymptomatic Pancreatic Enzyme Elevation

ICIs associated with pancreatic injury affect endocrine and exocrine functions of the pancreas (DM), and the effects may be asymptomatic or symptomatic. Diagnosis of acute pancreatitis depends on identification of at least 2 of the following features: (1) severe epigastric pain often radiating to the back; (2) elevated serum lipase/amylase levels (at least three times the upper normal limit); and (3) characteristic findings of acute pancreatitis on abdominal imaging. Imaging (CT, MRI, and PET/CT) of ICIs-associated pancreatitis shows any of the following: (1) new focal or diffuse pancreatic enlargement; (2) decreased enhancement and surrounding fat stranding without a focal lesion suspicious for metastasis; and (3) diffuse increased FDG uptake (62). Elevations in amylase/lipase are graded using the Common Terminology Criteria for Adverse Events (CTCAE 5.0) where by Grade 1 represents amylase or lipase levels ≤ 1.5 × upper limit of normal (ULN); Grade 2 represents levels > 1.5-2.0 × ULN; Grade 3represents levels >2.0 - 5.0 × ULN and Grade 4 represents levels > 5.0 × ULN. Notably, pancreatitis is defined by grading system whereby Grade 2 represents asymptomatic enzyme elevation or radiologic findings only; Grade 3 represents severe pain; vomiting; medical intervention indicated (e.g., analgesia, nutritional support); Grade 4 represents life-threatening consequences; urgent intervention indicated and Grade 5 represents cases resulting in death (57).

In a recent meta-analysis, incidence of asymptomatic elevation in pancreatic enzymes after ICIs treatment was 2.7% (211/7702), whereas incidence of grade 2 pancreatitis was 1.9% (150/7702). Furthermore, most patients with pancreatic injury and treated with ICIs show slight increase in amylase and lipase levels. However, patients have no symptoms of pancreatitis and present no radiographic abnormalities for pancreatic glands (58). Similar observations have been reported in other studies. Michot et al. explored elevated serum lipase with a grade of ≥2 related to an anti-PD1 or anti-PD-L1 treatment and its outcomes. They reported that most patients (71%, 15/21) who underwent anti-PD1 or anti-PD-L1 treatment presented with an asymptomatic increase in serum lipase levels, with a peak incidence in 0.4 months to 11.4 months (median=2.8 months, around 5 treatment cycles) after initiation of treatment. Moreover, approximately 14% (3/21) of patients developed typical acute pancreatitis (59).

The relationship between asymptomatically elevated amylase/lipase levels and pancreatitis is not clear. Friedman et al. enrolled 119 patients with melanoma treated with nivolumab + ipilimumab to a study. The findings from the study showed that 20% of these patients manifested grade ≥ 3 amylase elevations, 6.3% had grade ≥ 3 lipase elevations, 20% had increased levels of both enzymes, and 1.7% developed immune-related pancreatitis (60). Therefore, other nonimmune-mediated causes of asymptomatically elevated pancreatic enzyme levels should be explored. For instance, this increase may be related to T cell-mediated inflammation present in other organs, a metastatic disease, or renal failure, and may not be related to pancreatic inflammation (61, 63). Pancreatic enzyme monitoring in asymptomatic patients is not recommended as a routine procedure unless for cases where pancreatitis is suspected. NCCN guidelines state that treatment with ICIs can be continued for patients with slight increase in amylase and lipase levels (at least 3 times the upper normal limit) if pancreatitis is excluded (73).

## ICIs Associated With Pancreatitis

ICIs associated with pancreatitis (ICIs-P) are extremely rare irAEs, making diagnosis a clinical challenge. As previously described, diagnosis of ICIs-P requires at least 2 criteria among the significant symptoms of pancreatitis, including radiographic evidence, and changes in laboratory data. George et al. evaluated 33 trials in a meta-analysis and reported that incidence of pancreatitis in the CTLA-4 group was higher compared with that of the PD-1 group (4% vs. 1%). Moreover, incidence of grade 2 pancreatitis in CTLA-4-PD-1 combination group was 10.6%, significantly higher compared with that of mono-immunotherapy (58).

Kohlmann et al. reported a case of ICIs-P within the first 4 months of immunotherapy. The patient manifested a belt-shaped epigastric pain 106 days after immunotherapy initiation. Laboratory findings showed that serum lipase and serum amylase levels increased to 394.2 and 318 U/l, respectively. Pancreatic computed tomography showed edematous swelling within the tail of the pancreas. Therefore, ICIs-P was diagnosed based on the aforementioned criteria, immunotherapy was immediately halted, and the patient was administered withmethylprednisolone (1.3 mg/kg, total 128 mg). The symptoms gradually improved, and glucocorticosteroid dose was reduced. When the dosage was reduced to 8 mg, clinical symptoms relapsed, and serum lipase levels increased again (>600 U/l). Therefore, the patient received long-term treatment (6.5 months) of high-dose systemic methylprednisolone with significant slow tapering until clinical symptoms were cleared and normal levels of serum pancreatic enzymes achieved. Therefore, management of ICIs-P requires a high initial dose and slow tapering of glucocorticosteroid dose (64).

Pathophysiological features underlying ICIs-P remain elusive. Similar to other irAEs, mechanisms can be thourgh inflammatory responses in the pancreas after ICIs administration. A few studies report on functional T cell activation induced by ICIs treatment. Immunohistochemical analysis shows that CD3+ T lymphocytes densely infiltrate pancreatic islets in “healthy” (nontumoral) areas, thereby increasing the ratio of CD8+/CD4+ T lymphocytes in peritumoral areas. These findings imply that immune T cell infiltrates might be the prevalent cytotoxic components of ICIs treatment (65). Furthermore, dense CD8+, TIA1+, and granzyme B+ lymphoid infiltration are present within a biopsied lesion as shown by immunohistochemical analysis (66).

NCCN guidelines for management of ICIs-P state that when potential symptoms of ICIs-P appear, laboratory tests and abdominal imaging should be performed. Once the diagnosis of ICIs-P is confirmed, hospital admission is recommended, however, other management approaches depend on grading. For grade 2 pancreatitis (moderate), treatment with ICIs should be discontinued, and 0.5–1 mg/kg/day prednisone/methylprednisolone should be administered until symptoms improve to grade ≤1. After achieving grade ≤1, the dose should be tapered for 4–6 weeks, and IV hydration should be administered. For grade 3–4 of pancreatitis (severe and life-threatening), immunotherapy should be permanently discontinued, and treatment with a double daily dose of glucocorticosteroids rather than moderate grade and IV fluids should be started (73).

## ICIs Associated With Pancreatic Exocrine Insufficiency

Pancreatic exocrine insufficiency (PEI) is different from the first 2 types of pancreatic exocrine injury. Cancer patients under ICIs treatment might manifest weight loss despite their good appetite, irregular stools, steatorrhea, and abdominal pain, a condition called ICIs-PEI. Chronic pancreatitis and pancreatic surgery are the main causes of PEI, whereas immunotherapy does not commonly cause PEI. Prasanna et al. reported a case of a patient who developed isolated ICIs-PEI associated with pembrolizumab therapy. In this study, a 77-year-old male patient who underwent cutaneous melanoma resection from his left ear, was administered with pembrolizumab (2 mg/kg, every 3 weeks) after 4 years due to pulmonary metastases. After 16 months of treatment, the patient presented with explosive diarrhea and passing “stools like oil.” His CRP level was high, however lipase and amylase levels were normal. Colonoscopy and gastroscopy did not show any evidence of colitis. with the patients was suspected to have potential immune-related colitis, therefore, he was given prednisolone (empirical medication), shich showed no clinical response. A slight decrease in the fecal pancreatic elastase-1 level (<200 μg/g, moderate PEI) and a repeat test carried out 3 months later showed significantly lower level of pancreatic elastase-1 (<15 μg/g, severe PEI) compared with the level observed before. The patient was diagnosed with exocrine pancreatic insufficiency. Therefore, he was subjected to pancreatic enzyme replacement therapy (PERT), and his symptoms were progressively alleviated within 7–10 days (67).

Activated and increased CD8+ T cells were attributed to immunotherapy infiltrate inside and around the pancreas which resulted in damage of pancreatic cells. This phenomenon decreased the number of pancreatic ductal and acinar cells (exocrine pancreas) and resulted in pancreatic atrophy. These changes decrease secretion of pancreatic enzymes and affect release of bicarbonate, water, and enzymes into the duodenum. Furthermore, symptoms associated with food digestion (specifically fat malabsorption) are observed. Although direct methods are the gold standard for diagnosing ICIs-PEI, fecal pancreatic elastase-1 test an indirect test (100% sensitivity and 93% specificity) is commonly used (74). Pancreatin (pancreatin enteric-coated capsules) is used to increase mixing of chyme and pancreatic enzymes and for compensation of insufficient pancreatic enzymes, thus promoting nutrient uptake.

## Conclusion

Immunotherapy-associated pancreatic adverse events result in metabolic and nutritional disorders and are classified into pancreatic endocrine injury and exocrine insufficiency. Activation of T lymphocytes densely infiltrates in and around the pancreatic islets, destroys exocrine and endocrine pancreatic tissues thus causing ICIs-DM, asymptomatic pancreatic enzyme elevation, ICIs-P, and ICIs-PEI. Therefore, timely patient and physician education, clinical evaluations (systemic physical examination and appropriate laboratory tests) before each dose of immunotherapy, familiarity with ICIs-associated pancreatic injury characteristics by the clinicians, appropriate protocol of irAE management immunotherapy, self-surveillance, and outpatient follow-up are vital aspects affecting patient outcomes.

## Data Availability Statement

The original contributions presented in the study are included in the article/supplementary material, further inquiries can be directed to the corresponding author/s.

## Author Contributions

All authors contributed to the article and approved the submitted version.

## Funding

This research was sponsored by the Natural Science Foundation of China (NSFC), No. 51675356; the Key Research and Development Projects in Sichuan Province (No. 2020YFS0261); the Key Research and Development Projects in Sichuan Province (No. 2019YFS0043); and the 1·3·5 Project for Disciplines of Excellence–Clinical Research Incubation Project (ZY2017302), West China Hospital, Sichuan University.

## Conflict of Interest

The authors declare that the research was conducted in the absence of any commercial or financial relationships that could be construed as a potential conflict of interest.

## Abbreviations

ICIs, Immune checkpoint inhibitors; PD-1, Programmed death 1; PD-L1, Programmed death-ligand 1; CTLA-4, Cytotoxic T-lymphocyte antigen 4; irAEs, Immune-related adverse events; DM, Diabetes mellitus; Type 2 DM, Type 2 diabetes mellitus; F1DM, Fulminant type 1 diabetes; DKA, Diabetic ketoacidosis; GAD, Glutamic acid decarboxylase; Treg, T regulatory cells; HLA, Human leukocyte antigen; CRP, C-reactive protein; AGL, Acquired generalized lipodystrophy; ICIs-P, Immune checkpoint inhibitors associated with pancreatitis; PEI, Pancreatic exocrine insufficiency; SCC Jaw, Sarcomatoid squamous cell carcinoma of the Jaw; NSCLC, Non-small cell lung cancer; SCLC, Small-cell lung cancer; RCC, Renal cell carcinoma; MCC, Merkel cell carcinoma; HNSCC, Head and neck squamous cell carcinoma; HCC, Hepatocellular carcinoma; NR, Not reported; IA-2, Islet-associated antigen-2; IAA, Insulin autoantibodies; ZnT8, Zinc transporter 8; ICA, Islet cell antibody; CTCAE, Common Terminology Criteria for Adverse Events; ULN, Upper limit of normal.
